# Biochemical and biological evaluation of L-glutaminase from *Aspergillus tamarii* AUMC 10198 via solid-state fermentation

**DOI:** 10.1186/s12934-025-02802-0

**Published:** 2025-08-04

**Authors:** Ghada A. Youssef, Maii S. Zaid, Amany S. Youssef, Samy El-Aassar

**Affiliations:** Botany and Microbiology Department, Faculty of Science, Alexandria University, Alexandria, 21511 Egypt

**Keywords:** L-glutaminase, Media optimization, Enzyme purification and characterization, Antimicrobial activity

## Abstract

**Introduction:**

Fungal L-glutaminase has recently attracted growing interest due to its potential applications in medical therapy and biotechnology. This study aimed to develop a cost-effective bioprocess for L-glutaminase production using agricultural by-products under solid-state fermentation (SSF). Several fungal isolates were screened for extracellular L-glutaminase production, and the native isolated strain *Aspergillus tamarii* AUMC 10198 was identified as a potent high-yield producer. Process parameters influencing enzyme production were systematically optimized using a one-variable-at-a-time (OVAT) approach. The enzyme was subsequently purified through a three-step procedure and characterized for its biochemical properties. Notably, the purified L-glutaminase also exhibited antimicrobial activity, suggesting potential therapeutic applications.

**Results:**

The native fungus *Aspergillus tamarii* AUMC 10198, registered under GenBank accession number OQ976977, was identified as a potent producer of L-glutaminase under solid-state fermentation (SSF) using wheat bran as the solid substrate. The solid-state yield of L-glutaminase exhibited a 3.20-fold increase in comparison to the unoptimized state. L-glutaminase produced by *Aspergillus tamarii* AUMC 10198 was purified through three successive steps, leading to a 12.90-fold enhancement in enzyme activity. As a result of the purification process, the final enzyme recovery was 18.45%. The isolated L-glutaminase exhibited optimal activity at a pH of 8, a temperature of 45 °C, and partial stability up to 60 °C, as determined by characterization. The purified L-glutaminase exhibited a Vmax of 10.10 U/ml and a km of 0.28 mg/ml when glutamine was used as the substrate. The metal ions Fe^2+^, Ca^2+^, K^+^, Mg^2+^, and Na^+^ of 0.01 M concentration exhibited notable enzyme-activating effects, leading to an increase in L-glutaminase activity. The molecular mass was estimated to be approximately 62 kDa by SDS-PAGE. The produced enzyme showed notable antimicrobial activity, with the strongest effect against *Staphylococcus aureus* (36.80 ± 1.20 mm), followed by *Bacillus subtilis* (30.40 ± 0.60 mm), while the weakest inhibition was observed against *Pseudomonas aeruginosa* (12.80 ± 1.20 mm); moderate antifungal activity was also recorded highlighting its potential for broad therapeutic and pharmaceutical applications.

**Conclusion:**

This study highlights the remarkable properties of L-glutaminase produced by the native potent fungal isolate *Aspergillus tamarii* AUMC 10198, underscoring its significant potential for industrial applications and pharmaceutical drug development.

## Background

An enzyme that catalyzes the deamination of L-glutamine to L-glutamic acid and ammonia is glutamine amidohydrolase (EC 3.5.1.2), also referred to as glutaminase [1, 2]. Microbial glutaminases are efficient for use in commercial industries due to their rapid and cost-effective production methods, straightforward purification processes, and diverse applications [3]. The significance of L-glutaminases as enzymes with substantial commercial applications in the pharmaceutical and agricultural sectors has been underscored in recent publications [4, 5]. L-glutaminase, a green chemotherapeutic agent that inhibits the proliferation of numerous carcinoma cell lines and thereby impedes their development by depriving them of their essential amino acid (glutamine). Glutamine deficiency may induce metabolic starvation in cancer cells, inhibiting their proliferation, and thus represents a promising approach for anti-cancer therapy in the medical field [6–9]. Moreover, L-glutaminases are key agents in the biocontrol of microbial pathogens and exhibit promising biological activities with potential applications in the healthcare sector [10, 11]. Glutamic acid is produced through the hydrolysis of L-glutamine, the most essential amino acid in food production. Enzymatic deamidation by protein-glutaminase can promote the physical and chemical features of plant-based protein foods and improve their texture and stability as alternative protein sources [12]. L-glutaminase plays a crucial role in improving the flavor and aroma of traditional oriental fermented foods by converting glutamine into glutamate a compound known for its rich, umami taste, similar to the flavor provided by monosodium glutamate (MSG) [13]. This process obviates the necessity for monosodium glutamate (MSG) in food production. A considerable number of fermented foods, including Eastern condiments and soy sauce, acquire a robust, palatable, and umami taste due to the accumulation of L-glutamic acid [14, 15]. In addition, it is used to reduce the formation of acrylamide (probable human carcinogen) in fried foods, which typically occurs at high cooking temperatures. This reduction occurs indirectly by modulating the amino acid composition and altering the reaction conditions that favor acrylamide formation at high cooking temperatures [16]. Furthermore, L-glutaminase has been employed in the development of glutamine biosensors to detect glutamine concentrations in mammalian cell cultures, the biosynthesis of numerous nitrogenous metabolic intermediates at the cellular level, and the production of the nutraceutical theanine [17–20].

Microbial organisms that generate glutaminases include actinomycetes, fungi, yeasts, and bacteria [21, 22]. Fungi are the primary producers of extracellular hydrolytic enzymes. The environmentally friendly and sustainable properties of fungal enzymes have been the subject of extensive investigation and implementation in numerous industries, including agriculture, medicine, bioremediation, and industrial processing. These fungal enzymes are highly productive and stable and have low extraction and purification costs [23–25]. The significance of fungal glutaminases is due to their potential biological functions in different industrial and agricultural applications. One of the most important aspects is the production of a variety of fungicides with potential antifungal activities that play an impact on crop protection [26, 27]. Specific fungal species, including *Candida*,* Saccharomyces*,* Trichoderma*,* Aspergillus*,* Penicillium*,* Fusarium*, and a few endophytic species, are capable of producing glutaminase [28, 29]. Marine fungi are well known for the production of extracellular enzymes due to their potential physiological properties under harsh conditions. Rao et al. reported efficient production of L-glutaminase from marine filamentous fungi (*Aspergillus*,* Penicillium*) and yeasts (*Pichia* sp.) [5].

Two distinct fermentation processes are utilized for enzyme synthesis: solid-state fermentation (SSF) and submerged fermentation (SmF) [29, 30]. SSF has higher production, lower energy usage, simpler operations, and better product consistency than traditional SmF [31, 32]. Agricultural by-products have significant potential for use as sustainable carbon and energy sources, which generates economic and environmental benefits [33]. In addition, the proximity of solid fermentation systems to the natural habitats of microorganisms enables them to secrete and synthesize a vast array of metabolites and enzymes [29, 34, 35]. The objective of this study was to explore innovative strategies for developing a cost-effective bioprocess for L-glutaminase production from a native novel fungal strain exhibiting efficient enzymatic properties. Particular focus was placed on the evaluation and optimization of critical process parameters under solid-state fermentation (SSF) conditions. Furthermore, the enzyme was purified and characterized, and its antimicrobial activity was subsequently assessed.

## Materials and methods

### Screening and qualitative isolation of L-glutaminase-producing fungi

Wheat bran, a lignocellulosic by-product with high nutritional value, various samples of wheat bran were purchased from different commercial marketplaces in Abis National Village, Alexandria, Egypt. The materials were air-dried, pulverized to an 80-micron powder, and stored at 4–5 °C prior to processing. The serial dilution technique (one g of each sample) was used to isolate fungi on modified Czapek Dox’s (CZD) agar medium [36, 37]. Each screened fungal colony observed after five days of incubation at 28 °C was purified and subsequently preserved on potato dextrose agar (PDA) medium at 4 °C until further analysis. A rapid plate assay was used to qualitatively evaluate of L-glutaminase production. The experiment was performed via modified CZD solid medium, which was composed of the following components in g/L: 2.0 g of sucrose, 0.5 g of KCl, 0.5 g of MgSO_4_. 7H_2_O, 1.0 g of KH_2_PO_4,_ 0.1 g of FeSO_4_. 7H_2_O, 0.1 g of ZnSO_4_, 10.0 g of L-glutamine, and 20.0 g of agar at a pH of 6.0. The medium was supplemented with 0.009% (v/v) phenol red dye (Sigma-Aldrich, Saint Louis, Missouri (USA)) as an indicator. A stock dye solution was prepared by mixing 2.5 mL of a 3% (w/v) phenol red solution in ethanol and adjusting the pH to 7, as described by Gulati et al. [38]. A control medium devoid of dye was employed. The selection process focused on the most expansive and profound pink color zone encircling the colonies, the mean value of the zone diameters in triplicate was calculated. The positive isolated colonies were purified and maintained as slant cultures under refrigerated conditions. The isolate selected for further quantitative analysis was chosen based on its positive L-glutaminase production.

### Quantitative estimation of L-glutaminase

A qualitative evaluation was conducted to select the four fungal isolates (F-S1, F-S2, F-S3, and F-S4) that demonstrated the highest measurable L-glutaminase activity based on the plate assay results. Enzyme production was quantified using the submerged cultivation technique. One milliliter of spore suspension containing 1.5 × 10^7^ spores/mL was inoculated into a 250 mL Erlenmeyer flask. A fungal suspension was prepared from seven-day-old cultures grown on PDA slants and inoculated into 50 mL of modified Czapek-Dox broth supplemented with L-glutamine as the sole source of carbon and nitrogen. The cultures were incubated at 30 °C for 5 days under submerged fermentation (SmF) conditions on a rotary shaker at 150 rpm. After incubation, the culture broth was centrifuged at 4 °C for 15 min at 5000 rpm (Chilspin CR4, MSE Centrifuges Ltd., England). The resulting clear supernatant was collected as the crude enzyme extract and stored at 4 °C until further use.

### Assay of L-glutaminase

In accordance with the methodology described by Imada et al. L-glutaminase activity was assessed via the direct Nesslerization technique Imada et al. method [39, 40]. One milliliter of the crude enzyme was mixed with one milliliter of 40 mM L-glutamine, which was utilized as the substrate, in citrate-phosphate buffer (0.1 M, pH 7). Following incubation of the reaction mixture at 30 °C for 30 min, the reaction was terminated by the addition of 0.5 mL of 1.5 M trichloroacetic acid. To quantify the precipitated protein, 0.1 mL of the resulting solution was mixed with 3.7 mL of distilled water, followed by the addition of 0.2 mL of Nessler’s reagent (Himedia, India). The mixture was centrifuged at 5000 rpm for five minutes, and the absorbance of the supernatant was measured at 450 nm using a spectrophotometer (Alpha 1102, Laxco, USA). A unit of L-glutaminase (U) was designated to represent the quantity of enzyme required to produce 1 µmol of ammonia per minute under ideal assay conditions.

### Protein estimation

The protein content of thecrude enzyme was determined colorimetrically according to the method described by Lowry et al. with bovine serum albumin serving as a standard [41]. The protein concentration was expressed in mg/ml of crude enzyme.

#### Morphological characterization and molecular identification

At the Mycological Center, Assiut University, Assiut, Egypt (A.U.M.C.), An efficient L-glutaminase-producing fungal isolate was identified by combining taxonomic keys with morphological and reproductive characteristics [42, 43]. The genomic DNA of the sample under investigation was isolated, purified, and analyzed for molecular identification in accordance with the methodology described by Moubasher et al. [44]. Using universal primers ITS 4 (5’- TCC TCC GCT TAT TGA TAT GC- 3’) and ITS 1 (5’- TCC GTA GGT GAA CCT GCG G- 3’) produced by SolGent Co. in Yuseong-Gu, Daejeon, South Korea, the internal transcribed spacer (ITS) region was amplified using polymerase chain reaction (PCR), as described by Bagewadi et al. [45]. To examine the obtained sequences, the National Center for Biotechnology (NCBI) BLAST research tool was used (blast.ncbi.nlm.nih.gov). To construct the phylogenetic tree, 35 sequences from closely related *Aspergillus* (section Flavi) species in the GenBank database were aligned. Maximum likelihood (ML) models and maximum parsimony (MP) analyses were performed via version 10.2.6 of MEGA X [46]. The robustness of the sparsest-packed trees was assessed through the utilization of 1000 bootstrap replications [47].

### Solid-state fermentation and culture conditions

Quantitative analysis of L-glutaminase was conducted via the solid-state fermentation (SSF) method. A comprehensive survey of commercial markets in Abis National Village, Alexandria, Egypt, was carried out to collect various locally available agricultural waste materials, including wheat bran, soybean, sugarcane bagasse, and ground corn grains. The agro-waste substrates were maintained under aseptic conditions to prevent microbial contamination. The potential of these agro-industrial residues to enhance L-glutaminase production by *Aspergillus tamarii* was systematically evaluated. The substrate was mechanically dried at 50 °C to constant moisture, then ground using a mortar and pestle to a particle size of 500–1000 μm [48, 49]. Subsequently, 10 g of each substrate fraction were transferred into 250 mL Erlenmeyer flasks. Twenty mL of 0.01 M phosphate buffer pH 7.4 were added to moisten each flask [50]. After autoclaving at 121 °C for 20 min the flasks were inoculated with 2 ml of the spore suspension (1.5 × 10^7^ spores/mL) obtained from new slants (7-day old culture) of *Aspergillus tamarii*. The inoculated containers were incubated at 30 °C for five days under static conditions. In accordance with the modified methodology described by Kashyap et al. [51], crude L-glutaminase was extracted from the fermented solid culture by rotary shaking at 200 rpm for 20 min, followed by centrifugation at 1500 rpm for 20 min at 4 °C. The extraction was carried out using 50 mL of 0.1 M phosphate buffer (pH 7.0) as the solvent. The protein content and enzyme activity of the cell-free supernatant were evaluated. The mean ± standard deviation values are reported following the three repetitions of each experiment.

### Optimization of various parameters for L-glutaminase production under solid-state fermentation

The impact of various culture factors on L-glutaminase production by the most potent strain of *Aspergillus tamarii* during SSF was investigated by researchers employing a one-variable-at-a-time approach (OVAT). The effects of different culture parameters on L-glutaminase synthesis were investigated. These parameters included inoculum size (1.0–5.0% v/v), temperature (25–40 °C), incubation time (three to nine days), moisture content (0.25–4%), and pH ranging from 4.0 to 9.0 (1 N HCl or 1 N NaOH). Three replicates were conducted for each experiment [52].

### Purification of L-glutaminase

Crude L-glutaminase obtained from *Aspergillus tamarii* AUMC 10198 cultures grown under optimized conditions was purified through a three-step process involving ethanol precipitation, ion-exchange chromatography, and gel filtration chromatography. The partial purification of L-glutaminase was achieved through fractional precipitation using extremely absolute ethanol (4 °C). Cold crude enzyme and cold ethanol were combined in equal parts with gentle agitation and left at 4 °C for 20 min. The precipitated fraction was obtained by centrifugation at 5000 rpm and 4 °C for 15 min. The supernatant was subjected to ethanol precipitation, gradually saturated up to 90%. Precipitate fractions were collected at 30%, 50%, 70%, and 90% saturation levels. This fractional precipitation step was followed by overnight dialysis of the collected precipitates against 0.01 M phosphate buffer (pH 8.0) at 4 °C. The collected enzyme fractions were analyzed for total protein content and L-glutaminase activity [53]. Seven ml of the partially active ethanol fraction (70%) was loaded onto a DEAE Sephadex A-50 (2.5 cm × 30 cm) ion-exchange column pre-equilibrated with 0.1 M NaCl in a 0.05 M acetate buffer (pH 5.2). The enzyme elution was conducted at a flow rate of 30 ml/h. The entire quantity was collected at 4 °C. The total protein content and L-glutaminase activity of the active fractions were evaluated subsequent to their collection, pooling, and concentration. Sephadex G-100 (2 cm × 28 cm) was applied to load the active fractions of highest specific activity that generated in the previous stage. At a flow rate of 60 mL/h, the substance was eluted and brought to equilibrium utilizing the identical buffer. Protein absorbance was measured at 280 nm, and 5 mL of the most active fractions exhibiting the highest L-glutaminase activity were pooled, concentrated by dialysis against distilled water, lyophilized, and stored at − 20 °C for subsequent characterization of the purified enzyme.

### Properties of the purified L-glutaminase

#### Effect of substrate concentration on glutaminase activity

The optimal substrate concentration for the experiment was determined by performing separate incubations with purified L-glutaminase and different concentrations of L-glutamine (as a substrate) in the assay mixture (ranging from 0.2 to 2.0 mg/ml) under ideal conditions.

#### Kinetics parameters

To determine the kinetic parameters (maximal velocity (Vmax) and Michaelis-Menten constant (Km)) of the purified L-glutaminase, standard assay conditions were used to measure the reaction velocities at various concentrations of L-glutamine (0.2-2.0 mg/ml). The apparent km and Vmax values were obtained from the Lineweaver-Burk plot [54], which establishes a relationship between 1/Vmax and 1/S (reciprocal values), via the Michaelis-Menten equation,

$$ ~{\text{V}}_{0} = {\text{V}}_{{{\text{max}}}} \left[ {\text{S}} \right]{\text{ }}/{\text{ K}}_{{\text{m}}} + \left[ {\text{S}} \right] $$

In contrast, where V_0_ represents the initial velocity of the reaction, Vmax denotes the maximal velocity, S is the concentration of the substrate, and Km represents a constant.

#### Effects of pH, temperature, and thermal stability on glutaminase activity

The purified enzyme was subjected to different pH values ranging from 3.6 to 9.6 in acetate buffer (0.05 M; pH 3.6–5.4), phosphate buffer (0.05 M; pH 5.6-8.0), and sodium carbonate buffer (0.05 M; pH 8.6–10). The mixture was incubated under optimal assay conditions to establish the optimal pH for L-glutaminase activity. To investigate the effect of temperature on enzyme activity, the reaction mixture was incubated at temperatures ranging from 30 to 60 °C for 30 min at the optimal pH. To determine the thermostability of L-glutaminase, the enzyme was preincubated for 15, 30, or 60, min at temperatures of 50, 60, or 70 °C, respectively, in the absence of a substrate. Residual enzyme activity was subsequently assessed after chilling.

#### Effect of metal ions on glutaminase activity

The activity of purified L-glutaminase was investigated using a variety of metal ions at a concentration of 0.01 M: Na^+^ (NaCl), K^+^ (KCl), Ca^2+^ (CaCl_2_), Mg^2+^ (MgSO_4_.7H_2_O), Ba^2+^ (Bacl_2_), Cd^2 +^ (CdCl_2_), Cu^2+^ (CuSO_4_), Zn^2+^ (Zn (CH_3_COO)_2_), andFe^2+^ (FeSO_4_∙7 H_2_O). In the test mixture, each enzyme mixture was incubated with metal ions for 30 min at room temperature in accordance with the recommended modified assay protocol [55]. The estimated residual activity was compared to the 100% activity of the control group, which received no additives.

#### Molecular weight determination

The purity and molecular weight of the purified L-glutaminase were determined using sodium dodecyl sulfate-polyacrylamide gel electrophoresis (SDS-PAGE), performed with a 10% separating acrylamide gel, following the method described by Laemmli [56]. Gels were stained with Coomassie Brilliant Blue R-250.

#### Antimicrobial assay for purified L-glutaminase

The antimicrobial efficacy of the purified L-glutaminase was determined via the agar well diffusion technique [57] against six microbial pathogens kindly provided by the Microbiological laboratory at the Department of Microbiology, Faculty of Science, Helwan University, Egypt. *Bacillus subtilis* NRRL B-543 and *Staphylococcus aureus* ATCC 25923were used as Gram + ve bacterial indicators, and *Escherichia coli* ATCC 25,922 and *Pseudomonas aeruginosa* ATCC 27,853 were used as Gram -ve bacterial indicators. Moreover, *Candida albicans* ATCC 20,231 and *Aspergillus flavus* were used as fungal indicators. Bacterial pathogens were grown on Mueller-Hinton agar (MHA) at 35 °C for 1 day and fungal strains were grown on potato dextrose agar at 27 °C for 3 days. Muller-Hinton agar plates were swabbed with 100 µL (0.5 McFarland standards) of each strain of pathogenic organism. Wells approximately 6-mm in diameters were punctured aseptically in solid agar with a cork borer. One hundred microliters of the produced L-glutaminase was injected into each well. The injected plates were refrigerated for 2 h to permit the diffusion of the enzyme. The plates were subsequently incubated for 24 h at 37 ºC for bacteria and 5 days at 30 ºC for fungi. Gentamicin (10 µg/mL) was used as a positive control for bacteria and nystatin (100 U) was used for fungi [58]. The antimicrobial potential was evaluated by monitoring the mean diameter of the growth inhibition zones (mm) in triplicate.

### Statistical analysis

Data are presented as the mean value ± standard deviation. The means were calculated on the basis of information from three distinct investigations (*n* = 3). ANOVA was employed to compare the various groups that were the subject of the statistical investigation. The predetermined significance level was *p* < 0.05.

## Results and discussion

### Qualitative screening and quantitative estimation of L-glutaminase-producing fungi.

The L-glutaminase production potential of fourteen fungal isolates was initially screened using the rapid plate assay method. Out of the fourteen tested isolates, only four demonstrated positive results, indicated by the formation of a distinct pink to red halo surrounding the fungal colonies. This color change signifies the release of ammonia during L-glutamine hydrolysis, leading to a local pH increase and a visible shift in the phenol red indicator. The measurable pink zone diameters ranged from 8 to 32 mm. The appearance of such halos has been previously reported as a qualitative indicator of L-glutaminase activity by Balagurunathan et al. [59] and Nafisaturrahmah et al. [60]. The mean and standard deviation were estimated to show the results in Table 1. As determined qualitatively by the greatest mean diameter of the zone encircling the colonies on the plated agar, fungal isolate F-S1 showed the largest diameter zone (32.30 mm) and the lowest zone (7.88 mm) was detected for fungal isolate F-S3.

Quantitative estimation of L-glutaminase producing isolates was performed in triplicate via the agitated method with a submerged culture medium. The means and standard deviations were used to estimate the results in Table 1. The observed qualitative and quantitative values exhibited statistical dissimilarity when *p* < 0.05. The L-glutaminase activity of the fungal isolates exhibited a broad spectrum, with values varying from 0.45 ± 0.0^d^ to 2.32 ± 0.2^a^ U/ml. The discernible discrepancy is illustrated by the small letters. On the basis of a substantially distinct estimate (*p* < 0.05), the F-S1 isolate exhibited the highest enzyme production (2.32 ± 0.2^a^ U/ml) as a glutamine hydrolyzer. The L-glutaminase activities of F-S2, F-S4, and F-S3 were as follows: 1.25 ± 0.1^b^, 0.83 ± 0.1^c^, and 0.45 ± 0.0^d^ U/ml, respectively. A meticulous evaluation employing molecular and morphological techniques revealed that the potent isolate (F-S1) exhibiting the greatest glutaminolytic activity was selected for subsequent investigations.

**Table 1:** Qualitative and quantitative screening of L-glutaminase producing fungal isolates

Isolate number	Qualitative screening Mean pink zone diameter (mm)	Protein content (mg/ml)	Quantitative estimation Enzyme activity (U/ml)	Dry weight (g/50 ml)
F-S1	32.30 ± 3.55^a^	1.14 ± 0.11^a^	2.32 ± 0.2^a^	0.56 ± 0.1^a^
F-S2	15.55 ± 2.18^b^	0.623 ± 0.1^b^	1.25 ± 0.1^b^	0.39 ± 0.0^b^
F-S3	7.88 ± 0.87^c^	0.35 ± 0.0^c^	0.45 ± 0.0^d^	0.21 ± 0.0^c^
F-S4	9.02 ± 1.26^c^	0.43 ± 0.0^c^	0.83 ± 0.1^c^	0.36 ± 0.0^b^

Different lowercase letters (^a, b,c, d^) indicate significant differences among the different studied groups at *p* < 0.05.

ANOVA was performed for comparisons between different groups

The values are the means ± standard deviations (*n* = 3)

### Morphological properties and molecular identification of potent L- glutaminase-producing isolate

**Fig. 1:**
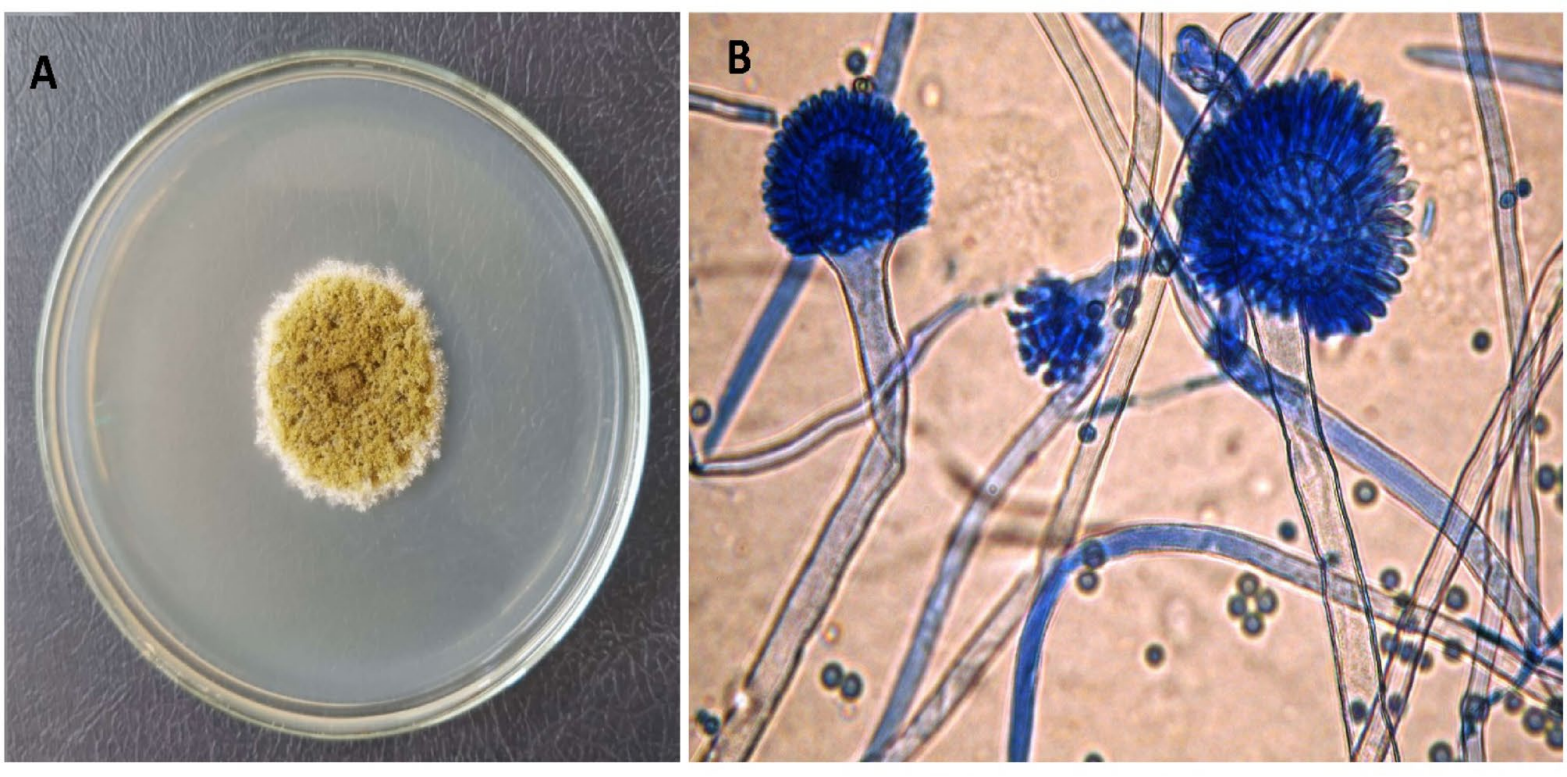
Morphological identification: *Aspergillus tamarii* (AUMC 10198) was cultured for seven days at 25 °C on Czapek agar media (**A**). A microscopic examination was performed via lactophenol cotton blue stain at X 1000 magnification (**B**)

The morphological identification of the potent glutaminase-producing fungal isolate (F-S1) *Aspergillus tamarii* AUMC 10198 was accomplished through phenotypic characterizations, assessment of reproductive structures, and comparisons with authentic isolates at the Mycological Center, Assiut University (AUMC), Assiut, Egypt. At the cultural level, colonies of *Aspergillus tamarii* (AUMC 10198) grown on Czapek agar ultimately grew to a diameter of 5–6 cm after seven days at 25 °C.The conidial region exhibited a subdued greenish-yellow hue with a white edge; the reverse region lacked any exudates or pigmentation (Fig. 1A). For microscopic examination (Fig. 1B), the lactophenol cotton blue-stained conidiophores were observed to be colorless, measuring a maximum of 1–2 mm in length and 8 μm in width. Additionally, they exhibited a grainy surface with an abrupt constriction of the wall at the base of the vehicle. The conidial head exhibited a loose radiating pattern and harbored sizable vesicles ranging in diameter from 30 μm. These vesicles are globose to subglobose in shape and have thin walls. When adhered to phialides that are loosely packed, they measure 8.5–11.5 × 5.0–6.0 μm. Large heads generally possess metulae measuring 10–15 × 4–8 μm in dimension, whereas small heads lack such features. Mature conidia are globose, bounded by chains that are conspicuously roughened, and range in diameter from 4.2 to 6.6 μm. In contrast, young conidia are cylindrical to pyriform.

**Fig. 2:**
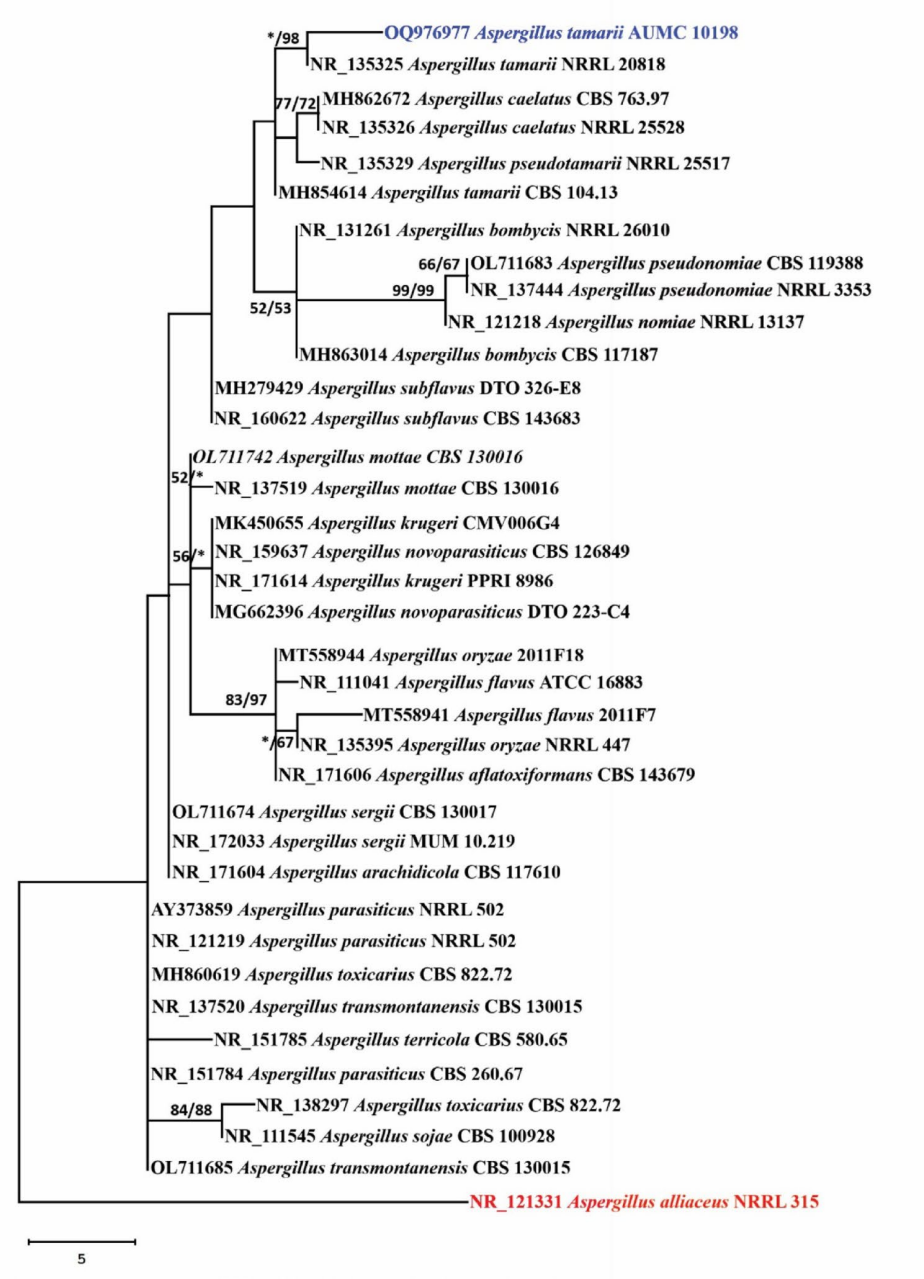
A phylogenetic tree of *Aspergillus tamarii* AUMC 10198 constructed from the ITS sequence in comparison to that in GenBank belonging to *Aspergillus*: section Flavi sequences that are closely related. This sequence is the *Aspergillus tamarii* blue sequence, designated with the accession number OQ976977. The bootstrap support values (calculated from 1000 replications) for ML/MP that are equal to or greater than 50% are displayed in close proximity to each node. In the ML/MP bootstraps, the symbol (*) denotes a significance level below 50%. The red *Aspergillus alliaceus* NRRL 315 constitutes the root system of the tree

A BLAST analysis was conducted to compare the extracted ITS sequences of the target strain with those that were previously archived in the NCBI Nucleotide Sequence Database. *Aspergillus tamarii* CBS 104.13, which has a GenBank accession number of MH854614 and 591 out of 597 identities (98.99%), was the most closely related match. The sequence, designated with accession number OQ976977, has been successfully submitted to the GenBank database. As shown in Fig. 2, the species was identified as *Aspergillus tamarii* AUMC 10198. The ITS region of *Trichosporon asahii* ChL11 isolate exhibited ≥ 98% sequence identity with members of the *Trichosporon* genus [61]. The nucleotide sequence datasets were compared with those of *Aspergillus* sp. ALAA-2000 [17].

### Solid-state fermentation (SSF) for L- glutaminase production

**Fig. 3:**
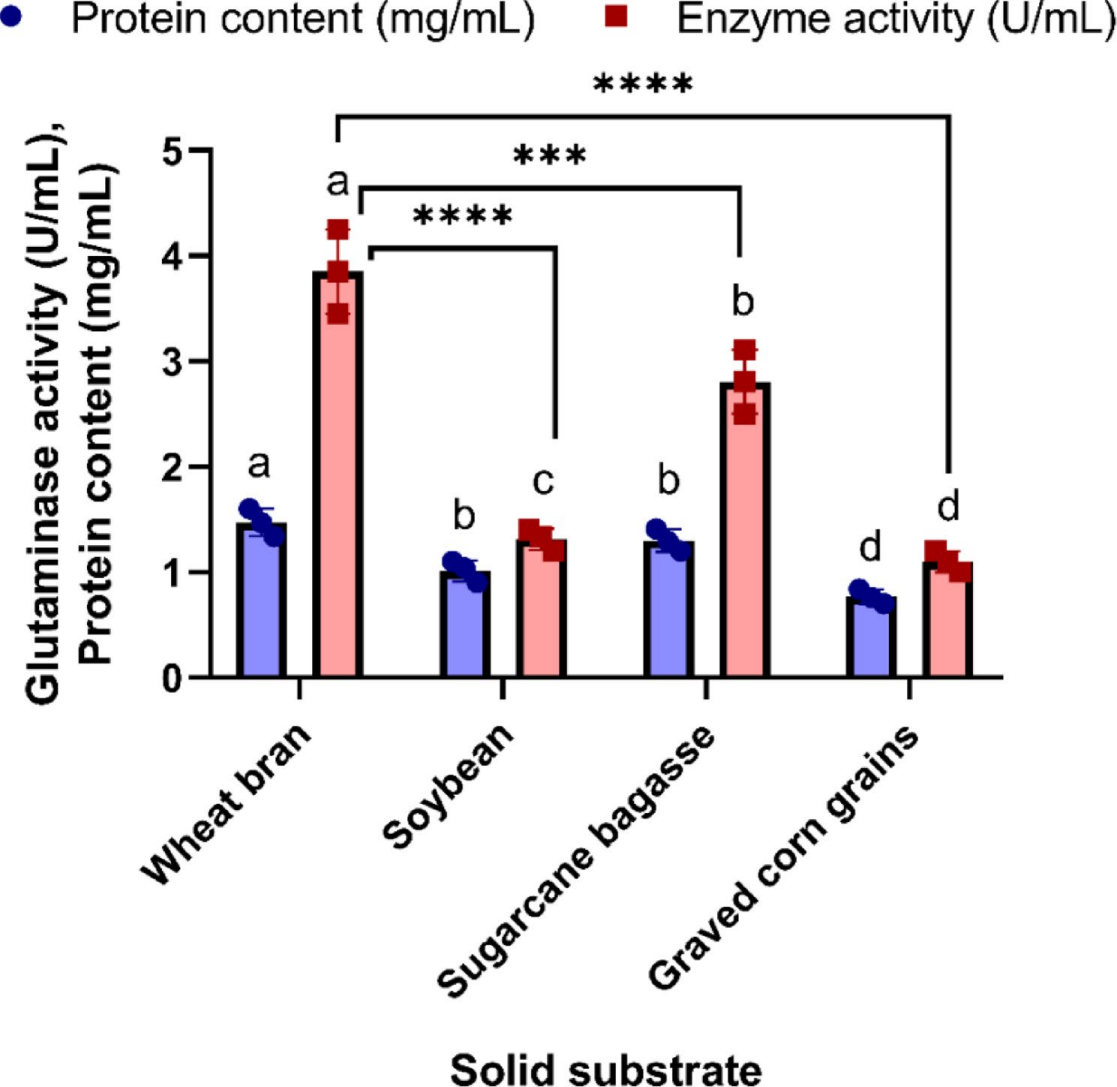
Screening of various solid substrates for L-glutaminase production by *Aspergillus tamarii* (F-S1) for five days at 30 °C under solid-state fermentation conditions. Data are presented as mean ± SD (*n* = 3). Statistical significance: ****p < 0.0001; ***p < 0.001. Different lowercase letters indicate significant differences between means at *p* < 0.05

Solid-state fermentation (SSF) is a sustainable and environmentally friendly approach that uses abundant agro-industrial by-products as cost-effective raw materials. The choice of an optimal solid substrate plays a crucial role in maximizing fermentation efficiency. The production of L-glutaminase varied depending on the type of substrate used. The particle size of a substrate used in solid-state fermentation (SSF) has a significant influence on microbial growth and enzyme production [48]. Selecting an appropriate particle size range is crucial for maintaining a balance between aeration, moisture retention, and substrate accessibility. The 500–1000 μm range was found to provide optimal conditions for microbial growth and L-glutaminase production [49]. As illustrated in Fig. 3, wheat bran proved to be the most effective solid substrate compared to sugarcane bagasse, soybean, and graved corn grains. It exhibited the highest specific activity of 2.61 ± 0.26 U/mg protein and enzyme activity of 3.85 ± 0.4 U/mL. Based on the current findings, there were significant differences in the specific activities of wheat bran, sugarcane bagasse, and graved corn grains, which recorded values of 2.61 ± 0.26^a^, 2.17 ± 0.18^b^, and 1.44 ± 0.13^c^ U/mg protein, respectively.

Our findings are consistent with those of Soren et al. who reported that wheat bran is the optimal substrate for maximal L-glutaminase production [62]. Similarly, wheat bran was identified as the most effective among seven solid by-products for enhancing L-asparaginase activity by *Fusarium solani* AUMC 8615 [63]. El-Sayed also demonstrated that wheat bran is the most suitable solid substrate for inducing L-glutaminase production by *Trichoderma koningii* [64]. The high nutritional content of wheat bran likely promotes fungal growth and spore formation [51]. Additionally, its resistance to particle aggregation contributes to improved mechanical efficiency during the fermentation process [65, 66].

### Optimization of SSF parameters for L-glutaminase production via the OVAT approach

#### Incubation time

**Fig. 4:**
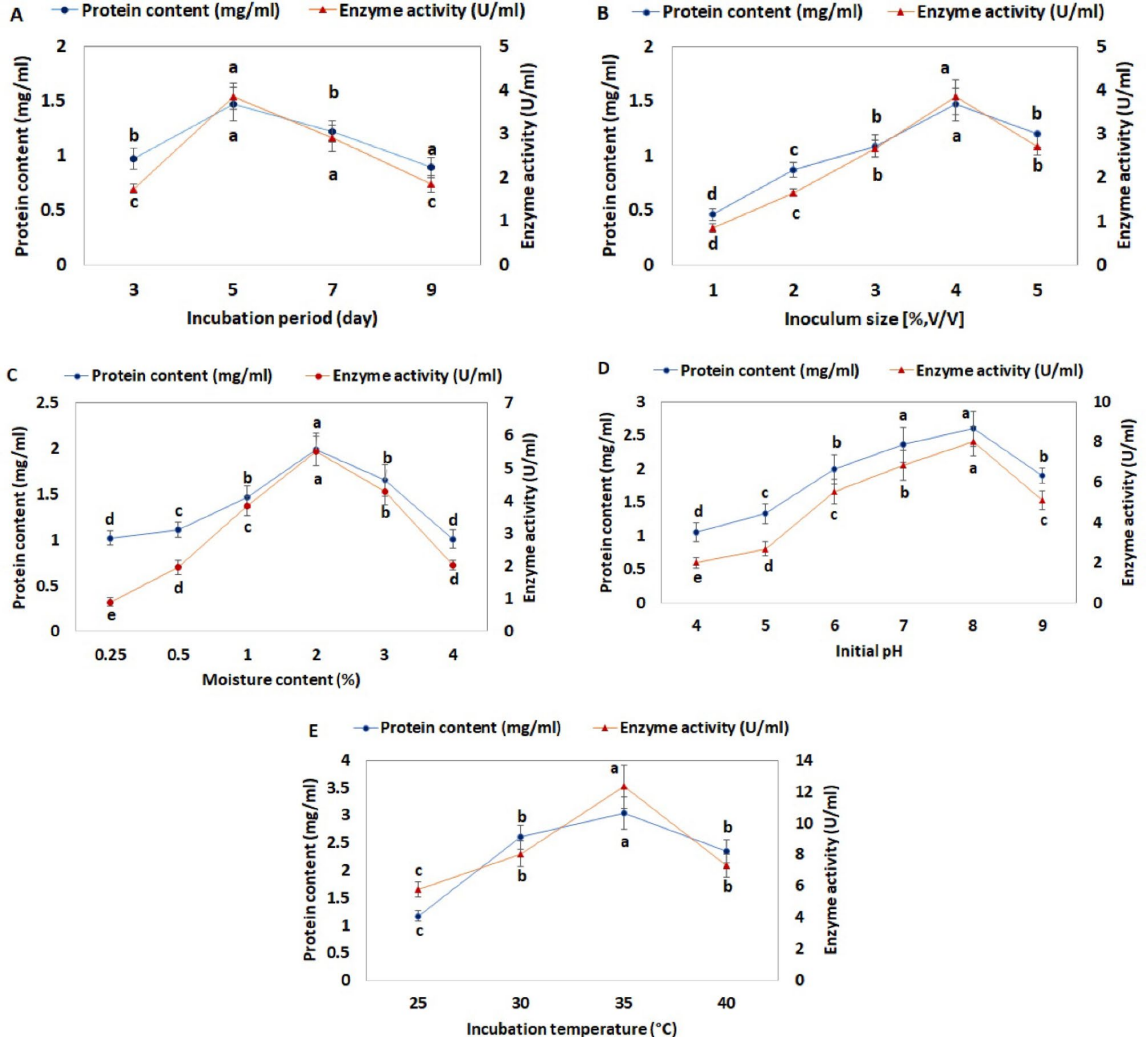
Optimization of various parameters for L-glutaminase production under solid-state fermentation by *Aspergillus tamarii* AUMC 10198: effects of incubation period (**A**), inoculum size (**B**), moisture content (**C**), initial pH (**D**), and incubation temperature (**E**). Data are presented as mean ± SD (*n* = 3), different lowercase letters indicate significant differences between means at *p* < 0.05

As shown in Fig. 4A, the duration of fermentation significantly influenced enzyme yield. The maximum L-glutaminase production was recorded on the fifth day of incubation, reaching 3.85 ± 0.3^a^ U/mL following a marked increase. Enzyme activity remained relatively stable with no significant difference observed on the third and ninth days, yielding 1.74 ± 0.1^c^ and 1.85 ± 0.2^c^ U/mL, respectively. Extending the fermentation period beyond five days led to a notable decline in enzyme production, likely due to enzyme degradation over time [67, 68]. The reduction in enzymatic activity was attributed to nutrient depletion and the accumulation of toxic metabolites in the fermentation medium, both of which impose physiological stress on microbial cells and adversely affect enzyme stability and activity [69], in addition to the presence of proteolytic enzymes that can degrade L-glutaminase [51]. Similarly, the optimal incubation period for L-glutaminase production was 5 days by *Streptomyces enissocaesilis* DMQ-24 [70]. On the other hand, Bagewadi et al. observed peak L-glutaminase activity on the fourth day [10]. In contrast, for most fungal strains, the optimal period for L-glutaminase synthesis typically falls between the fifth and seventh days of incubation, corresponding to the logarithmic growth phase [64, 71].

#### Inoculum size

The impact of varying inoculum concentrations (1–5% v/v) on L-glutaminase production during fermentation were evaluated, as illustrated in Fig. 4B. Maximum enzyme activity was observed at a 4% inoculum level (2 mL/flask), yielding 3.85 ± 0.4^a^ U/mL. *Trichoderma koningii* exhibited its highest L-glutaminase activity at the same inoculum volume (2 mL), in agreement with our findings [71]. Enzyme activity declined significantly when the inoculum size deviated from this optimal level. Abdel-Hamid et al. reported that *Fusarium oxysporum* showed peak enzyme activity at a 3% inoculum concentration under SSF conditions [72]. For accurate comparison, it is crucial to standardize the inoculum based on spore concentration rather than volume or percentage. A lower inoculum size can result in prolonged growth lag, reduced enzyme yield, and inadequate biomass formation, thereby hindering substrate utilization and target product synthesis [73]. Conversely, an excessively high inoculum volume can deplete essential nutrients and suppress enzyme production [51]. Moreover, from an industrial scalability perspective, this parameter can be proportionally adjusted for larger bioreactors, ensuring consistent performance while maintaining cost-effectiveness and operational feasibility.

#### Moisture content

Moisture content is a critical factor influencing fungal growth, as it regulates metabolic activity and significantly impacts product formation [74, 75]. As shown in Fig. 4C, the highest L-glutaminase activity was observed at an initial moisture content of 2%, yielding 5.52 ± 0.5a U/mL with a specific activity of 2.77±0.25^a^ U/mg protein. Beyond this optimal moisture level, enzyme production declined markedly. Excessive moisture limits gaseous exchange, restricts aeration, and promotes substrate particle aggregation, thereby reducing porosity and impairing microbial efficiency [76]. These findings are consistent with previous studies on L-glucoamylase synthesis by *Aspergillus niger* [77].

#### Hydrogen ion concentration

The concentration of hydrogen ions (pH) in the fermentation medium is a key factor influencing microbial growth, metabolic activity, and enzyme production [78, 79]. L-glutaminase production is generally optimized within a pH range of 6.0 to 8.0 in most microorganisms, as noted by Sathish and Prakasham [80]. In the current study, enzyme activity increased with rising initial pH levels, reaching a peak of 8.04 ± 0. 7^a^ U/mL at pH 8.0, indicating that a slightly alkaline environment is optimal for L-glutaminase synthesis (Fig. 4D). These results are in agreement with the findings of Mostafa et al. and Dueramae et al. who reported that *Halomonas meridiana* and *Tetragenococcus muriaticus* FF5302 exhibited maximal L-glutaminase production at pH 8.0 [22, 81]. Similarly, Rajesh et al. observed the highest enzyme activity by *Aspergillus terreus* at the same pH [82]. Compared to the optimal pH, L-glutaminase activity decreased significantly, retaining only 66.67% of its activity at acidic pH 5.0 and 36.32% at alkaline pH 9.0. However, *Aspergillus tamarii* demonstrated the ability to grow across a broad pH range (6–9), showing no significant difference in activity between pH 6 (5.52 ± 0.6^c^) and pH 9 (5.12 ± 0.5^c^). According to Lakshmi and Jaya, the optimal pH for L-glutaminase production by *Aspergillus oryzae* NCIM 1212 was pH 7 [83]. Additionally, *Aspergillus terreus* MTCC 1782 produced the highest level of L-asparaginase at pH 8 under SSF conditions [84]. Interestingly, *Beauveria* sp. presented dual pH optima for L-glutaminase production at pH 6.0 and 9.0 under solid-state fermentation [31].

#### Incubation temperature

The highest production of L-glutaminase (12.33 ± 1.4^a^ U/mL) was observed at an incubation temperature of 35 °C in the solid-state culture medium (Fig. 4E). Gomaa reported the maximum glutaminase activity of 40.80 U/mL of halophilic *Bacillus* sp. DV2-37 at 37 °C [85]. In contrast, *Trichoderma koningii* achieved peak L-glutaminase production at 30 °C [64]. In the current study, a significant decrease in enzyme activity to 7.3 ± 0.7^b^ U/mL was observed at 40 °C, likely caused by enzyme denaturation at temperatures outside the optimal range [68]. Overall, as shown in Fig. 4, L-glutaminase production under solid-state fermentation increased by approximately 3.20-fold compared to pre-optimization conditions. Similarly, *Aspergillus terreus* ZHG2 demonstrated a 3.23-fold enhancement in L-glutaminase activity relative to an unoptimized medium [49]. Furthermore, optimization efforts under SSF yielded a maximum productivity of 703.8 U/g dry substrate, representing a 3.8-fold increase [86].

### L-glutaminase purification

#### Ethanol fractionation

L-glutaminase was partially purified by ethanol precipitation at 70% saturation Table 2. It exhibited a total activity of 303.98 U, a specific activity of 13.10 U/mg protein, an enzyme recovery of 24.65%, and a purification fold of 3.23.

**Table 2:** Ethanol precipitation of *Aspergillus tamarii* AUMC 10198 L-glutaminase

Ethanol Concentration (%)	Protein content (mg/ml)	Total protein (mg)	Recovered protein (%)	Glutaminase activity (U/ml)	Total activity unit (U)	Recovered activity (%)	Specific activity (U/mg protein)	Purification (fold)
Culture extract	3.04	304	100	12.33	1233	100	4.06	1
30	1.31	13.1	4.31	13.98	139.8	11.33	10.67	2.63
50	1.69	16.95	5.58	19.83	198.3	16.09	11.70	2.88
70	2.32	23.20	7.63	30.39	303.98	24.65	13.10	3.23
90	0.53	5.30	1.74	4.12	41.2	3.34	7.77	1.91
Total			19.26			55.41		

#### Ion-exchange chromatography

**Fig. 5:**
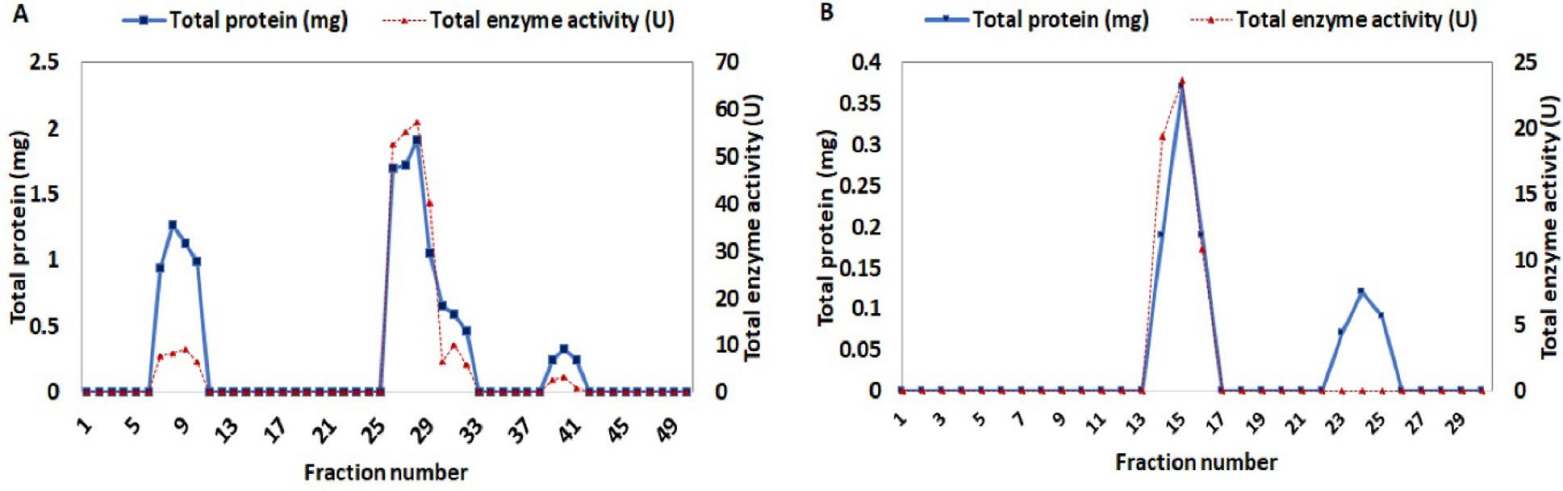
Purification stages of *Aspergillus tamarii* AUMC10198 L-glutaminase: A 0.1 M NaCl solution was introduced into a 0.05 M acetate buffer with a pH of 5.2 and a flow rate of 30 ml/h to calibrate a 2.5 cm × 30 cm Sephadex A-50 DEAE ion-exchange chromatography column (**A**). The 5 mL fractions were subsequently obtained by means of gel filtration through a Sephadex G-100 column measuring 2 cm × 28 cm with identical buffer and a flow rate of 60 mL/h (**B**)

The DEAE (Sephadex A-50) column was used in the subsequent purification step, yielding three protein peaks that corresponded to three L-glutaminase activity peaks were eluted at fractions 6–11, 25–30, and 38–42 (Fig. 5A). Protein binding is governed by factors such as the pH and ionic strength of the solution, the net charge of the protein molecule, and the binding capacity of the ion exchange resin [87]. Accordingly, the first peak, eluted in the early fractions, likely represents enzyme molecules that did not bind to the anion exchange resin due to their lower net negative charge under the applied buffer conditions. In contrast, the second and third peaks correspond to enzyme populations that interacted with the resin and were gradually eluted by increasing the ionic strength of the elution buffer. Among these, the second peak showed the highest L-glutaminase activity, indicating it contained the major active form of the enzyme. Specific activity increased 6.89-fold, rising from 13.10 to 27.99 U/mg protein, with a yield of 22.57%, as detailed in Table 3.

#### Gel filtration chromatography

The most active fractions obtained from ion-exchange chromatography using Sephadex A-50 were further purified by gel filtration chromatography on a Sephadex G-100 column. The elution profile revealed two protein peaks, including a sharp and distinct peak corresponding to L-glutaminase activity in fractions 13 to 17, as shown in Fig. 5B. These active fractions (5 mL) were pooled, concentrated, and stored at − 20 °C. The purified enzyme was then used for subsequent analyses to meet additional purity criteria.

An overview of the purification results is detailed in Table 3. The calculated values are as follows: 54.17 U/mg protein for specific activity, 227.50 (U) for total activity, 18.45% for final yield, and 12.90-fold purification compared with the crude enzyme. The L-glutaminase of *Aspergillus flavus* exhibited a specific activity of 613.30 U/mg and a yield of 51.11%; the purification procedure was multiplied by 12.47 [65]. The L-glutaminase obtained from *Aspergillus versicolor* Faesay4 was found to have a purification efficiency of 2.10 ± 3.18 and a specific activity of 398.79 ± 9.81 U/mg protein with an overall activity of 13.16 ± 22.76 units [40]. In contrast, *Penicillium brevicompactum* NRC 829 demonstrated 869.08 U/mg specific activity, 321.6 U total activity, 162.75-fold purification, and 48.21% yield on Sephadex G-200 [88]. In accordance with the findings of Ali et al. *Penicillium politans* NRC510 L-glutaminase presented the following values: a yield of 25%, a specific activity of 133 U/mg, and a purification level of 230-fold [89].

**Table 3:** Summary of the purification stages of *Aspergillus tamarii* AUMC 10198 L-glutaminase

Purification stage	Total Protein (mg)	Total activity unit (U)	Specific activity (U/mg protein)	Yield (%)	Purification (fold)
• Crude extract	304	1233	4.06	100	1
• Ethanol (70%)	23.20	303.98	13.10	24.65	3.23
• Ion exchange on DEAE-Sephadex A-50	9.94	278.25	27.99	22.57	6.89
• Gel filtration on Sephadex G-100	4.2	227.50	54.17	18.45	12.90

### Characterization of purified ***Aspergillus tamarii*** AUMC10198 L-glutaminase

**Fig. 6:**
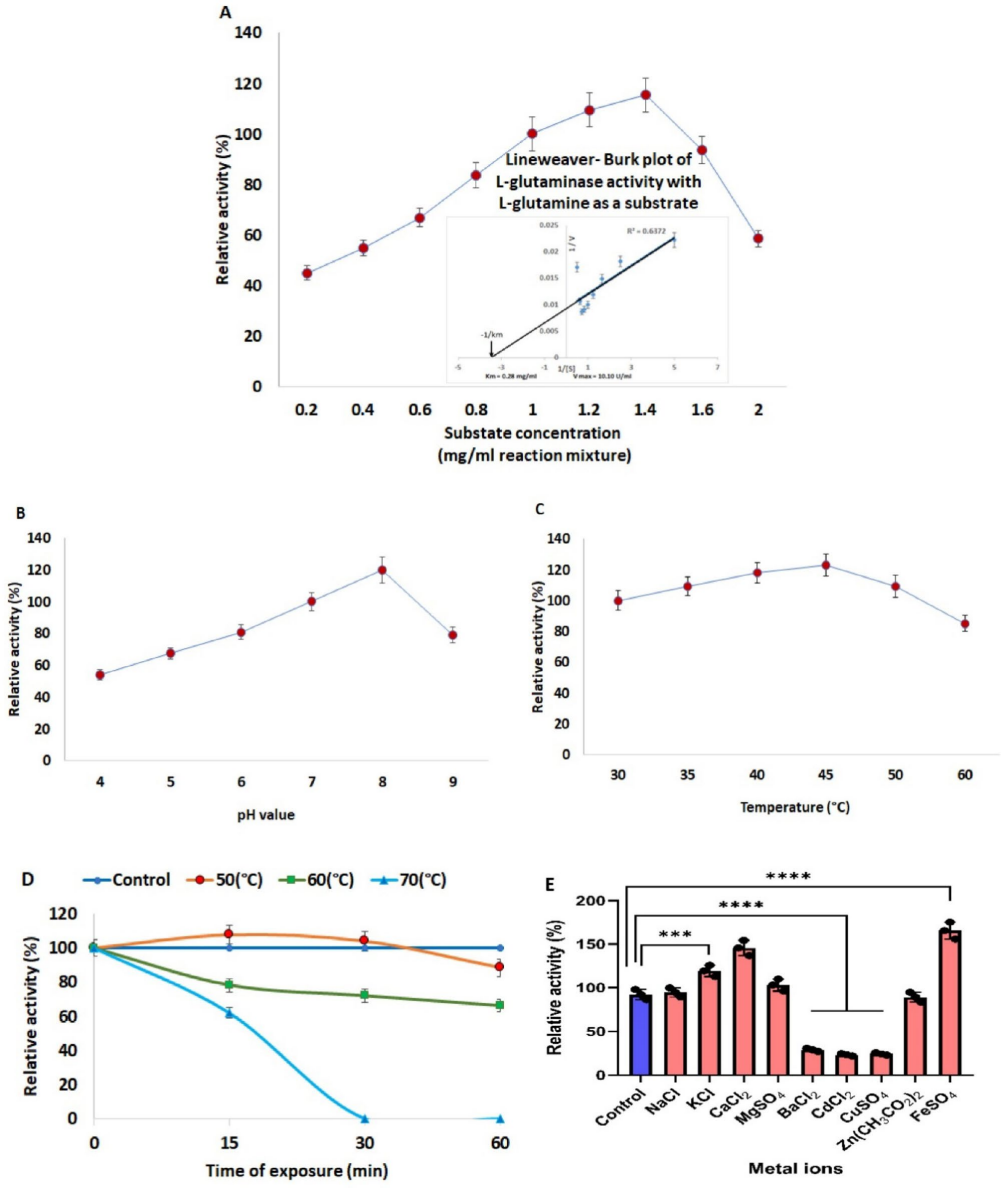
Characterization of *Aspergillus tamarii*AUMC10198 L-glutaminase: Lineweaver-Burk plot evaluation of the kinetic parameters (Km and Vmax) with respect to substrate concentration (**A**), pH (**B**), reaction temperature (**C**), thermal stability (**D**), and metal ions (**E**). Data are presented as mean ± SD (*n* = 3) of three independent replicates. Statistical significance: ****p < 0.0001; ***p < 0.001

Several parameters including the substrate concentration, kinetic parameters (km and Vmax), pH level, reaction temperature, thermal stability, and the effect of metal ions, were considered when determining the L-glutaminase activity of *Aspergillus tamarii* AUMC10198 under the test conditions (Fig. 6).

#### Effect of substrate concentration and estimation of the kinetic parameters (Km and Vmax)

The effects of varying L-glutamine concentrations on the activity of purified L-glutaminase are shown in Fig. 6A. Substrate concentrations ranging from 0.2 to 2.0 mg/mL was tested. Enzyme activity increased steadily with substrate concentration, reaching a maximum relative activity of 115.38% at 1.4 mg/mL L-glutamine. However, further increases in substrate concentration led to a decline in enzyme activity. Ahmed et al. reported the highest L-glutaminase activity for marine *Aspergillus* sp. ALAA-2000 at a substrate concentration of 4.38 mg/m [17]. Jayabalan et al. detected optimal activity of *Brevundimonas diminuta* MTCC 8486 L-glutaminase at 1% glutamine [90]. The enzyme’s strong affinity for L-glutamine is reflected by a low Km value of 0.28 mg/mL and a high Vmax of 10.10 U/mL, as depicted in Fig. 6A. Km values estimate the affinity of the enzyme for its substrate [91]. It is important to note that enzyme kinetic constants (Km and Vmax) are affected with different factors, such as the type and form of the enzyme (crude, semi purified, or purified), its biological origin, the substrate used, and the methodology of analysis [92]. In agreement with the current results, Singh and Banik reported that the great affinity of purified L-glutaminase to L-glutamine was detected at a small km (0.129 mmol) by *Bacillus cereus* MTCC 1305 [93]. *Penicillium brevicompactum* NRC829 exhibited the highest affinity for L-glutaminase activity at 1.66 mM L-glutamine, with a km value of 0.13 mM, as reported by Elshafei et al. [88]. On the other hand, Bagewadi et al. reported that *Streptomyces roseolus* ZKB1 L-glutaminase exhibited a Km of 13.89 ± 0.8 mM and a Vmax of 18.40 ± 1.5 U/mL, indicating Michaelis–Menten kinetics in their study [10].

#### Effects of pH, temperature, and thermal stability on glutaminase activity

As shown in Fig. 6B, the maximum relative activity of 119.73% was achieved at pH 8, indicating that the optimal pH range for L-glutaminase activity lies between neutral and mildly alkaline conditions (pH 7–8). These results are consistent with previous reports from *Debaryomyces* sp. and *Penicillium politans* NRC [1, 89]. Microbial glutaminases generally exhibit optimal activity under alkaline conditions [94]. In this study, enzyme activity significantly declined at both acidic (pH 4) and alkaline (pH 9) extremes. Optimal pH values for glutaminase activity have been reported around 8.0 and 8.5, respectively [88, 95]. It has been demonstrated that the effective pH range for glutaminase activity typically extends from 5.0 to 9.0 [96–98].

A clear relationship was observed between incubation temperature and glutaminase activity. The enzyme exhibited peak activity at 45 °C and retained over 85% of its activity at 60 °C (Fig. 6C). The decline in enzyme activity at higher temperatures is likely due to heat-induced denaturation of enzyme subunits [99]. These findings are supported by the study of Farag et al. which reported maximum L-glutaminase activity from marine *Aspergillus terreus* at a similar optimal temperature [100]. The thermostability of the purified enzyme was evident, as it retained approximately 88.59% and 66.29% of its activity after preincubation at 50 °C and 60 °C, respectively, for 60 min (Fig. 6D). However, a significant loss in activity approximately 38.11% was observed after 15 min of incubation at 70 °C, with complete inactivation occurring after 30 min at the same temperature. The efficiency of biotechnological processes is often constrained by issues related to enzyme stability and heat-induced deactivation, making the evaluation of these properties crucial for identifying enzymes suitable for industrial applications. Elshafei et al. reported that L-glutaminase purified from *Penicillium brevicompactum* NRC829 remained stable within the temperature range of 50–60 °C [88]. Similarly, *Aspergillus oryzae* L-glutaminase was stable at 45 °C but lost activity entirely at 55 °C [66].

#### Effect of metal ions (activators/inhibitors) on the activity of purified L-glutaminase

Metal ions play a vital role in enzyme activity by either donating or receiving electrons. The enzyme maintained over 100% of its initial activity when promoted by Fe^2+^, Ca^2+^, K^+^, Mg^2+^, and Na^+^ ions as shown in Fig. 6E. These metal ions can act as cofactors, facilitating the activation of the enzymatic reaction by promoting the formation of the enzyme–substrate complex and subsequently accelerating the release of reaction products [101]. The fold values of purified L-glutaminase for each of these metal ions were as follows: 1.80, 1.58, 1.29, 1.12, and 1.03. In contrast, Cd^2+^, Cu^2+^ and Ba^2+^ significantly impeded the activity of the enzyme. However, L-glutaminase activity was only slightly inhibited by Zn²⁺, while Cd²⁺ caused a further 75% reduction in enzyme activity. Husain et al. reported that the divalent and trivalent cations, Ca2+, Mg2+, Zn2+, Mn2+, and Fe3 + inhibited the enzyme activity [102]. *Aspergillus* sp. ALAA-2000 and *A. flavus* were capable of eliciting the activity of purified L-glutaminase when subjected to Na^+^ and Mn^2+^ ions [17, 65]. Conversely, Fe^2+^ exhibits moderate inhibitory activity against L-glutaminase from the marine *Bacillus subtilis* [103]. In general, the enhancing effect of metal ions can be attributed to their ability to stabilize the enzyme’s conformational structure and protect it from autoproteolysis and thermal denaturation [9]. Certain glutaminases have been reported to exhibit metalloenzyme-like properties, demonstrating increased catalytic activity in the presence of specific metal ions [1]. It has been demonstrated that metal ions can modulate the ionic interactions within the enzyme–substrate complex, leading to either stimulation or inhibition of enzymatic activity.

#### Estimation of the molecular weight of L-glutaminase by SDS–PAGE.

**Fig. 7:**
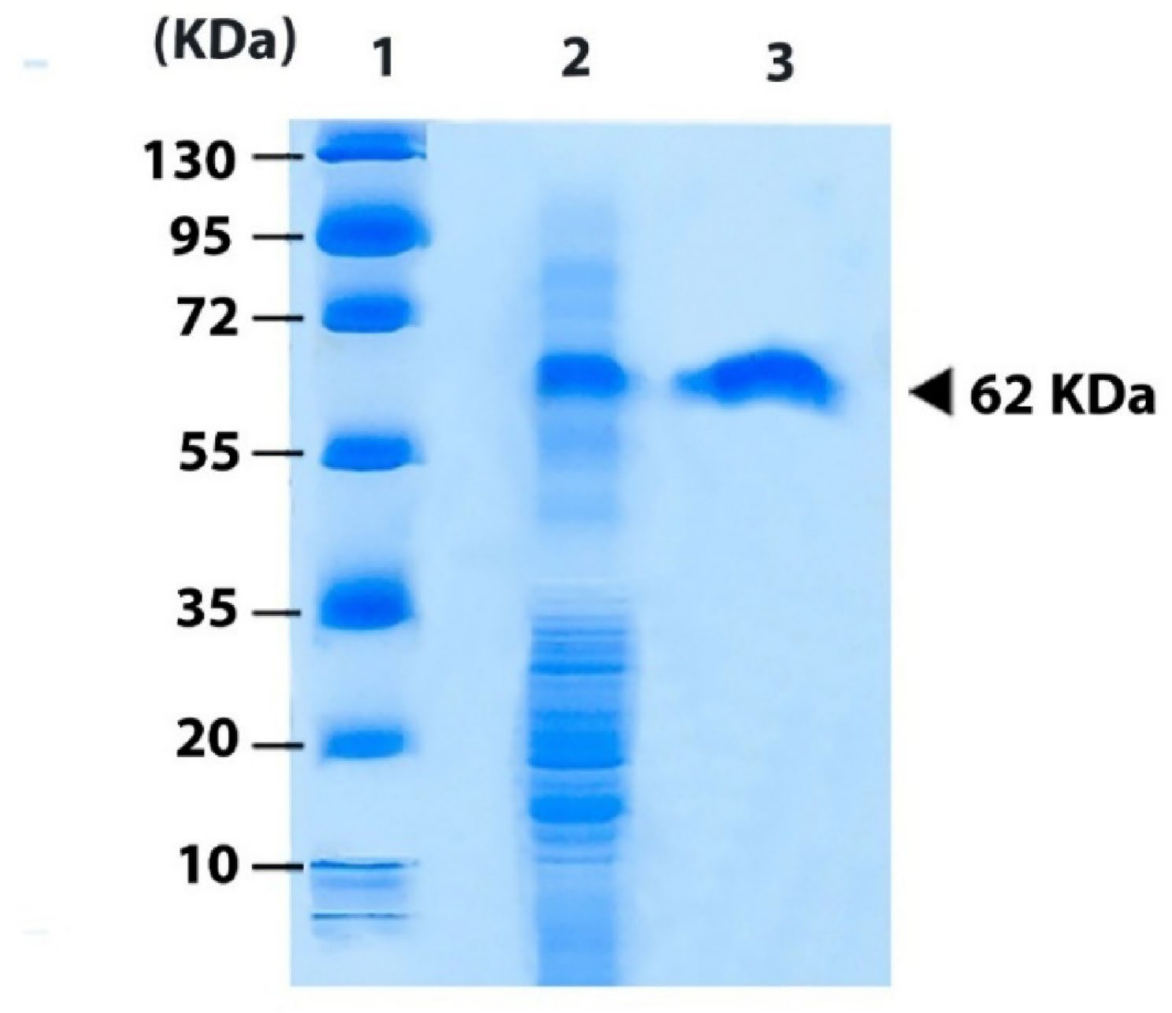
SDS-Polyacrylamide gel electrophoresis of the purified* Aspergillus tamarii* AUMC10198 L-glutaminase. Lane 1: Protein marker; Lane 2: Semi purified enzyme; Lane 3: Purified enzyme from Sephadex G-100

SDS-PAGE analysis assessed a single distinct protein band corresponding to an approximate molecular weight of 62 kDa (Fig. 7), suggesting that the L-glutaminase is a homogeneous protein. These results align with the findings of several researchers, including Elshafei et al. [88] and Bazaraa et al. [104], who reported that L-asparaginase and L-glutaminase purified from *Penicillium brevicompactum* NRC 829 and *Aspergillus oryzae* NRRL 32,567, respectively, exhibited single bands on SDS-PAGE, with estimated molecular weights of 94 kDa and 68 kDa. SDS-PAGE analysis of *Klebsiella pneumoniae* (AS KP 23) glutaminase indicated that the subunits of the purified enzyme had an estimated molecular weight of 97 kDa [105]. Although most fungal L-glutaminases are monomeric, some exhibit diverse oligomeric structures [88, 106]. Similarly, certain bacterial glutaminases can form dimeric or tetrameric forms [107]. The observed variations in molecular weight among different L-glutaminases are likely due to differences in the microbial sources and the intrinsic properties of the enzymes produced.

#### Antimicrobial activity of purified* Aspergillus tamarii* AUMC10198 L-glutaminase

L-glutaminase may have the potential to be an effective antimicrobial agent for preventing and inhibiting microbial growth by reducing the availability of glutamine (via converting glutamine to glutamate), a key nutrient for many pathogens. Glutamine plays a vital role in various metabolic processes, including cell proliferation, energy production [108]. The data in Table 4 show the antimicrobial efficiency of purified *Aspergillus tamarii* AUMC10198 L-glutaminase against several pathogenic indicators. The enzyme showed the highest antibacterial activity against *Staphylococcus aureus* ATCC 25,923 followed by *Bacillus subtilis* NRRL B-543 with mean inhibition zone diameters of 36.80 ± 1.20 mm and 30.40 ± 0.60 mm, respectively. The lowest zone of inhibition (12.80 ± 1.20 mm) was achieved against *Pseudomonas aeruginosa* ATCC 27,853. Marine *Streptomyces griseorubens* NAHE L-glutaminase which has potential antibacterial activity against Gram-positive bacteria comparable to that of Gram-ve bacteria [109]. According to another study, L-glutaminase exhibited the maximum antibacterial activity of 26 ± 0.3 mm against *Bacillus subtilis* and the lowest activity of 13 ± 0.4 mm against *Staphylococcus aureus* [10]. The antifungal activity of *Aspergillus tamarii* AUMC10198 L-glutaminase resulted in moderate inhibition zone diameters. In contrast, the antimicrobial impact of *Pseudomonas* sp. RAS123 L-glutaminase, which has no antimicrobial activity against the fungal pathogens *Aspergillus fumigatus* (RCMB 002008) and *Candida albicans* (RCMB 005003(1), ATCC 10231), was investigated [95].

**Table 4:** Antimicrobial efficiency of purified *Aspergillus tamarii* AUMC10198 L-glutaminase against various pathogenic indicators

Agent	Mean diameter of the growth inhibition zones (mm ± SD)					
	Bacterial indicators				Fungal indicators	
	Gram + ve		Gram -ve			
	S*. aureus* ATCC 25923	*B. subtilis* NRRL B-543	*E. coli* ATCC 25922	P*. aeruginosa* ATCC 27,853	*C. albicans* ATCC 20231	*A. flavus*
Purified L-glutaminase	36.80 ± 1.20	30.40 ± 0.60	15.30 ± 1.50	12.80 ± 1.20	22.40 ± 0.50	26.09 ± 1.20
Gentamicin (10 µg/mL)	28.20 ± 1.60	26.20 ± 0.30	20.20 ± 0.50	18.70 ± 0.10	0.0	0.0
Nystatin (100 U)	0.0	0.0	0.0	0.0	23.00 ± 1.40	18.06 ± 0.90

## Conclusion

The present study revealed that the local native strain *Aspergillus tamarii* AUMC10198 exhibits significant potential for L-glutaminase production, using wheat bran as an economical and environmentally sustainable substrate. Optimization using the One-Variable-at-a-Time (OVAT) approach led to a 3.20-fold increase in extracellular L-glutaminase yield compared to unoptimized conditions. Followingpurification, the enzyme activity showed a 12.90-fold enhancement relative to the crude extract. In addition, the L-glutaminase from *A. tamarii* AUMC10198 exhibited promising antimicrobial activity against various pathogenic organisms. These findings offer valuable insights into the biotechnological production of L- glutaminase from indigenous fungal strain *Aspergillus tamarii* AUMC10198. Future research will focus on conducting detailed antimicrobial assays to elucidate the mechanisms of inhibition and further investigate the molecular pathways underlying the enzyme’s chemotherapeutic potential, particularly in anticancer applications. The promising properties demonstrated in this study pave the way for the industrial exploitation of *A. tamarii* AUMC10198-derived L-glutaminase in both medical and biotechnological fields.

## Data Availability

No datasets were generated or analysed during the current study.
